# Evaluation of the effectiveness of the Indian government’s policies to strengthen health warning labels on smokeless tobacco products: findings from the 2010–2019 Tobacco Control Project India Surveys

**DOI:** 10.1136/tc-2023-058281

**Published:** 2024-01-12

**Authors:** Ian Holdroyd, Namrata Puntambekar, Pete Driezen, Shannon Gravely, Anne C K Quah, Steve Shaowei Xu, Prakash C Gupta, Geoffrey T Fong, Mangesh S Pednekar

**Affiliations:** 1 University of Cambridge School of Clinical Medicine, Cambridge, UK; 2 Healis Sekhsaria Institute for Public Health, Navi Mumbai, Maharashtra, India; 3 Department of Psychology, University of Waterloo, Waterloo, Ontario, Canada; 4 School of Public Health Sciences, University of Waterloo, Waterloo, Ontario, Canada; 5 Ontario Institute for Cancer Research, Toronto, Ontario, Canada

**Keywords:** Packaging and Labelling, Non-cigarette tobacco products, Public policy

## Abstract

**Background:**

Smokeless tobacco (SLT) packaging in India had a single symbolic (a scorpion) health warning label (HWL) in 2009 covering 40% of the front surface. In 2011, it was replaced with four pictorial images. In 2016, HWLs were enlarged to 85% on the front and back. This study aimed to assess the effectiveness of the old (symbolic and smaller images) and larger HWLs.

**Methods:**

Data were from the Tobacco Control Project India Survey and included respondents who used SLT in Wave 1 (2010–2011, n=5911), Wave 2 (2012–2013, n=5613) and Wave 3 (2018–2019, n=5636). Using a repeated-measures design, weighted logistic regression models assessed whether there were changes in seven HWL effectiveness measures within the domains of awareness, salience, cognitive and behavioural responses. A cohort design was employed to test whether HWL effectiveness in Waves 1 and 2 was associated with quitting SLT in Waves 2 and 3, respectively.

**Results:**

The 2011 HWL revision did not result in any significant changes in HWL effectiveness. There was no significant change in HWL awareness and salience after larger HWLs were introduced in 2016, but respondents were more likely to consider SLT health risks (Wave 2=17.9%, Wave 3=33.6%, p<0.001) and quitting SLT (Wave 2=18.9%, Wave 3=36.5, p<0.001). There was no change in HWLs stopping SLT use (Wave 2=36.6%, Wave 3=35.2%, p=0.829); however, respondents were more likely to avoid looking at HWLs (Wave 2=10.1%, Wave 3=40.2%, p<0.001). Effectiveness of older, symbolic and smaller pictorial HWLs was not associated with quitting SLT.

**Discussion:**

There was no significant change in HWL effectiveness following the revision from a symbolic to a pictorial image, but enlarging pictorial images resulted in some improved cognitive and behavioural effects. Results suggested wear-out of HWL salience and that the effectiveness of warnings depends on both their design and time since implementation.

**WHAT IS ALREADY KNOWN ON THIS TOPIC**

- HWL changes in 2011 were shown to not alter HWL effectiveness in a cohort of Indian people using smokeless tobacco (SLT).
- Larger size HWL increased HWL effectiveness in a range of global studies focusing on individuals using cigarettes.

**WHAT THIS STUDY ADDS**

- The first study investigating the effects of recent SLT HWL changes in India, when HWL size requirements were increased to 85% on tobacco packaging.
- We found that enlarging the pictorial images resulted in improved measures of cognitive and behavioural HWL effects.
- The study suggests that HWL wear-out may have resulted in the lack of effectiveness on awareness and salience measures.

**HOW THIS STUDY MIGHT AFFECT RESEARCH, PRACTICE OR POLICY**

- These findings support the actions of both the government of India and other countries to increase HWL size requirements on SLT packaging.
- The Indian government should consider a range of changes to counter the low level of salience. This could include regularly refreshing graphic image content and highlighting the benefits of quitting.
- This study uses a Tobacco Dependence Index, shown to be strongly predictive of quitting SLT, appropriate to the future study of SLT control in India.

## Introduction

 Smokeless tobacco (SLT) refers to a diverse range of tobacco-based products which are consumed by a process other than smoking, such as sniffing, chewing, keeping in the mouth or applying on teeth or gum.^1 2^ An estimated 300 million people use SLT globally.^1^ Like smoked tobacco, SLT carries significant health risks,^3 4^ which are especially high in Asia, the Middle East and Africa.^5^ SLT use has been linked to oral,^6^ pharyngeal,^2^ oesophageal,^6 7^ gastric^8^ and breast cancers.^9^ Globally in 2017, more than 258 000 deaths from ischaemic heart disease and 90 000 deaths from oral, pharyngeal and oesophageal cancers^2^ were attributable to SLT use. Over 85% of these deaths occurred in South Asia and Southeast Asia, where SLT use is highly prevalent.

Tobacco use remains a significant public health issue in India, with a prevalence of 28.6% reported in 2016–2017.^10^ Unlike many countries, SLT use exceeds smoked tobacco use: in 2016–2017, 21.4% of adults reported using SLT while 10.7% reported using smoked forms.^10^ As of 2014, over 70% of the world’s SLT users were from India,^1^ accounting for 70% of the global burden of SLT-related deaths due to ischaemic heart disease, oral cancer and a range of other cancers.^2^

### SLT and health warning labels in India

Since ratifying the WHO Framework Convention on Tobacco Control in February 2004, the Indian government implemented a range of policies attempting to reduce the use of both smoked tobacco and SLT. Multiple studies have summarised these policies and assessed India’s progress, identifying that the largest change has been seen in policies regarding tobacco health warning labels (HWLs).^11 12^ HWLs are an effective tobacco control measure aimed at communicating risks and encouraging tobacco cessation.^13^ The Indian parliament passed the Cigarettes and Other Tobacco Products Act which required that by February 2007 all tobacco products carried HWLs. Tobacco industry influence delayed this until May 2009.^14^ From May 2009 to November 2011, the symbolic image of a scorpion covered 40% of the front of tobacco packages.^14 15^ These HWLs were poorly understood and ineffective in changing health behaviours.^16 17^

These initial HWLs were replaced in December 2011 by four pictorial HWLs of the same size accompanied by either the text ‘TOBACCO KILLS’ or ‘SMOKING KILLS’. Cross-sectional evidence from people who smoke suggests that pictorial warnings had been noticed by respondents, were understandable and would help individuals to quit.^18 19^ However, this change did not result in a significant increase in HWL effectiveness in a cohort of people who used SLT.^20^ The 2011 HWLs were revised in 2013 to three pictorial HWLs of the same size and style; two images were the same and one was new.

India’s government subsequently legislated that from 1 April 2016, HWLs must cover at least 85% of tobacco packs—with at least a 60% pictorial and 25% textual warning. These changes were supported by global empirical evidence.^13^ For example, following the increased size of HWLs in Uruguay, people who used cigarettes reported increased awareness, salience, cognitive effects and behavioural effects of HWLs.^21^ In Australia, increased HWL size resulted in increased attention towards HWLs, in addition to increased thoughts of the harmful effects of smoking.^22^ Table 1 shows examples of HWLs in 2009, 2011 and 2016, in addition to the respective survey wave when the effectiveness of each group of HWLs was measured in this study.

**Table 1:** Description of health warning labels on smokeless tobacco (SLT) packaging in India from 2009 to 2016

Date of policy change	HWLs from May 2009	HWLs from December 2011	HWLs from April 2016
Summary of policy change	First HWLs mandated:Symbolic image must cover at least 40% of the front of SLT tobacco packaging	Change to a pictorial HWL:Pictorial image must cover at least 40% of the front of SLT tobacco packaging	Increase in size of a pictorial HWL:Pictorial image must cover at least 85% of the front and back of SLT tobacco packaging. Warnings must be at least 3.5 cm in width and 4 cm in height.
Example image	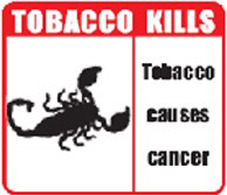	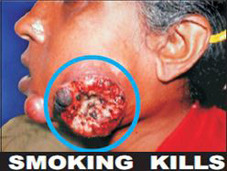	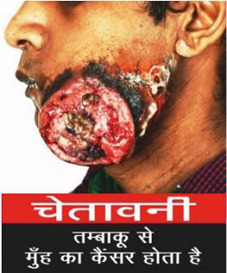
Wave when HWL’s effectiveness was measured	Wave 1: August 2010–October 2011	Wave 2: October 2012–September 2013	Wave 3: July 2018–July 2019

HWLs, Health Warning Labels.

Initial evidence suggests these changes may have had a positive impact. A cross-sectional study of individuals who reported using smoked tobacco or SLT and were attending outpatient department services at a tertiary care centre in Rishikesh in 2018–2019 found that 57.3% of respondents noticed the increased size of HWLs.^23^ A repeat cross-sectional study of two nationally representative cohorts of Indian people who used cigarettes found an increase in reporting noticing HWLs from 71.3% in 2009–2010 to 82.8% in 2016–2017, and an increase in reporting HWLs having an impact on quit intentions from 50.6% to 76.6%.^24^

To date, no study has examined the impact of the enlarged HWLs among people using SLT in India, which is important considering SLT’s significant public health burden and the different sociodemographic characteristics in users of smoked tobacco and SLT.^25^ On a global scale, while aforementioned studies have explored the effect of a change in HWL size,^21 26^ none were performed in a country with as large an HWL size requirement as India, nor did they investigate SLT users. Additionally, these studies were performed in more economically developed countries, with higher literacy rates and income. The association of HWL effectiveness and whether an individual who uses SLT in India quits tobacco remains unexplored.

A previous study that tested the effectiveness of changing SLT HWLs from symbolic to graphic images found that the change to graphic images did not lead to significant increases in the effectiveness of any HWL indicators among those who continued to use SLT products.^20^ Our current study builds on this study and addresses the aforementioned gaps in research, by including data collected following the increased HWL size requirements. By including seven wide-ranging measures, within the categories of HWL awareness, salience, cognitive effects and behavioural effects, this analysis offers a comprehensive assessment of Indian HWL effectiveness over a long period of time. The objectives of this study were to test (1) whether HWL changes in 2011 and 2016 resulted in a change in HWL effectiveness and (2) whether a measure of the effectiveness of older 2009 or 2011 HWLs predicted that an individual quit SLT.

## Methods

### Study design and procedure

Data for this study came from the Tobacco Control Project (TCP) India Survey, a prospective cohort study of individuals aged ≥15 years from urban or rural areas of four Indian states: Bihar, West Bengal, Madhya Pradesh and Maharashtra. To date, three survey rounds (waves) have been completed. Wave 1 was conducted from August 2010 and October 2011, Wave 2 from October 2012 to September 2013 and Wave 3 from July 2018 to July 2019.

A stratified multistage cluster sampling design was used to sample respondents. Those who completed the Wave 1 survey were invited again to complete the next surveys (Wave 2, Wave 3), and those who were lost to attrition were replaced by new respondents. In order to adjust for potential disproportionate selection of adults who used and did not tobacco in subgroups, enumeration and survey weights were calculated for each enumerated household and survey respondent. Respondents answered a face-to-face survey in their preferred language: Hindi, Marathi, Bengali or English. Fieldwork was conducted by trained field staff. A token of appreciation was presented to each respondent, a gift equivalent to US$3. Ethics clearance was granted by the University of Waterloo, Office of Research Ethics and the Healis-Sekhsaria Institute for Public Health, Institutional Ethics Committee. Further details about household enumeration in the four states, the study sampling design, the construction of sampling weights, the selection criteria for survey respondents in each household, and the response rates are provided in the TCP India Technical Reports.^27^

### Study sample

**Figure 1:**
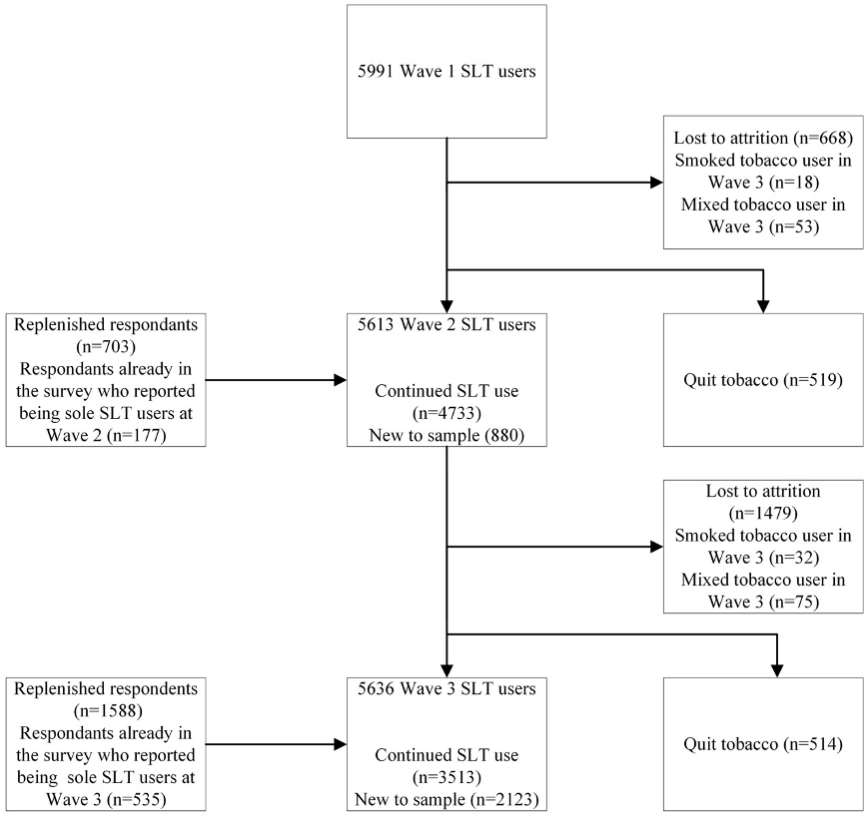
Study flow diagram. SLT, smokeless tobacco.

Respondents who exclusively used SLT at least once a month (ie, any SLT product such as Khaini, Gutka, Pan Masala, Mishri, Mawa, Gul, Bajjar and Gudhaku) and provided complete data for all sociodemographic measures and at least one set of outcome data were considered eligible for the current study. Two separate study samples were used. First, to investigate changes in HWL effectiveness, all individuals present in any wave meeting the inclusion criteria were selected. Second, to investigate factors predicting tobacco cessation, respondents who (1) were present in two successive waves; (2) reported exclusive SLT use in the initial wave; (3) were followed to the subsequent wave and (4) were aware of HWLs on SLT packaging at their initial wave were selected. Figure 1 shows a flowchart depicting the study sample at each wave.

### Measures

Sociodemographic characteristics were assessed using standard questions on sex, age, marital status, highest educational attainment, monthly household income and residential location (urban vs rural). Income and education were combined to form a composite indicator of socioeconomic status (SES).^28^ Respondents were classified into three discrete groups for both income and education. Income was grouped into ‘low’ (≤5000 Indian rupees)‘moderate’ (5001–15 000 Indian rupees) and ‘high’ (≥15 001 Indian rupees), and education was grouped into ‘low’ (up to middle school education), ‘moderate’ (completed secondary school) and ‘high’ (completed graduate, postgraduate or professional degree or above). Three SES groups were formed using these measures: ‘low’ (both income and education were low), ‘moderate’ (either income or education were low) or ‘high’ (neither income nor education were low). Respondents who answered only one item (n=534) were classed as the SES category of their answered item. SES data were not available for seven participants.

Three covariates controlled for respondents’ tobacco use behaviours and attitudes. (1) A Tobacco Dependence Index was calculated by addition of the score for the frequency of tobacco use per day (0=≤10, 1=11–20, 2=21–30, 3=≥31) and the score for the time between waking and first use (0=>60 min, 1=31–60 min, 2=6–30 min, 3=≤5 min). (2) Past-year quit attempts assessed whether respondents had tried to quit in the previous year (yes, no/don’t know). (3) Intentions to quit SLT assessed whether respondents planned to quit SLT (yes, no/don’t know).

### Outcome variables

Conceptual work and empirical studies have identified key indicators of HWL effectiveness, which have been employed in a range of studies across different countries.^26 29^ These key indicators were as follows:

1. *Awareness* was assessed with the question “As far as you know, do packages for ANY smokeless tobacco products in India have warning labels?” (yes vs no/don’t know).
2. *Salience* was assessed using two questions: “In the last 30 days, how often have you NOTICED warning labels on ANY packages of smokeless tobacco products?” (never, once in a while, often, whenever I use an SLT product) and “In the last 30 days, how often have you read or looked closely at the warning labels on packages of smokeless tobacco products?” (never, rarely, once in a while, often, regularly). Response options were dichotomised into “often/whenever I use a smokeless tobacco product” versus otherwise and ‘often/regularly’ versus otherwise, respectively.
3. *Cognitive responses* were assessed using two questions: “To what extent do the warning labels on packages of smokeless tobacco products make you more likely to think about the health risks of using them?” (not at all, a little, a lot) and “To what extent do warning labels on the packages make you more likely to quit using smokeless tobacco products?” (not at all, a little, a lot). Responses to both questions were dichotomised into ‘a lot’ versus otherwise.
4. *Behavioural responses* were assessed using two questions: “In the last 30 days, have the warning labels stopped you from using smokeless tobacco products when you were about to use one?” (never, a couple of times, once in a while, many times) and “In the last 30 days, have you made any effort to avoid looking at or thinking about the warning labels on packages of smokeless tobacco products?” (yes, no). Responses to the former question were dichotomised into ‘never’ versus at least a ‘couple of times’.

For analysis of change in each measure of HWL effectiveness, n=9 (“How often have you read or looked closely at the warning labels on packages of smokeless tobacco products?”) to n=185 (“To what extent do warning labels on the packages make you more likely to quit using smokeless tobacco products?”) respondents were excluded due to ‘don’t know’ responses.

### HWL impact index

A single measure of HWL effectiveness was used to analyse the effect of HWL impact on quitting SLT. This measure, called the Labels Impact Index (LII), was calculated using internationally validated methodology employed in previous ITC studies.^30 31^ The LII is a composite measure of four HWL effectiveness measures: noticing HWLs, HWLs causing thoughts of health risks, HWLs causing thoughts of quitting and HWLs causing an individual to forgo using tobacco. Original, non-dichotomised scores were standardised (z scores) and weighted before summing across all measures such that LII = (1*Noticing) + (2*thoughts) + (2*quitting) + (3*forgoing). Higher LII scores indicate a stronger HWL impact. LII scores are positively associated with a higher knowledge of health risks of tobacco, lower tobacco use, having stronger quit intentions and making quit attempts.^31 32^

### Statistical analysis

Unweighted descriptive statistics were used to characterise the sample of respondents according to sociodemographic measures and tobacco use behaviours and attitudes.

Logistic regression models, fit using generalised estimated equations (GEEs), were used to (1) estimate a weighted, adjusted percentage for each outcome measure prepolicy and postpolicy and (2) test differences in outcomes between prepolicy and postpolicy periods. Model outputs and SEs were used to estimate predictive margins (or adjusted percentages), marginal differences between waves, and CIs. All models adjusted for survey wave, age, sex, living in an urban area, marital status, SES, having made a quit attempt in the last year, intentions to quit and dependence. Sociodemographic measures were entered as time-invariant covariates and past-year quit attempts, intentions to quit and dependence were entered as time-varying covariates.

To assess the effectiveness of older HWLs predicting quitting, GEE models were also fit to examine the association between baseline HWL effectiveness (LII) and covariates in Waves 1 and 2 on the likelihood of quitting SLT in Waves 2 and 3, respectively. Respondents were included in the analysis if they were present in two consecutive waves and reported using SLT exclusively in the initial wave. The cessation outcome was defined as reporting having quit tobacco use at the subsequent survey (vs still using SLT exclusively). This model adjusted for the same covariates as listed above. A wave-by-LII interaction term was included to separately examine the effects of LII in Waves 1 and 2.

Models were estimated in SUDDAN (V.11.0.3) and accounted for the multistage sampling design, longitudinal sampling weights and repeated measures. Models were fit with a binomial distribution, logit link and an exchangeable working correlation. All p values were calculated with χ^2^ tests. A significant result was defined as p<0.05. A Bonferroni correction was applied to account for multiple comparisons.

## Results

### Respondent characteristics

Table 2 shows respondent’s unweighted descriptive statistics. At baseline, the majority of respondents were male (57.4%), lived in urban areas (73.4%) and were married (72.1%). During the study period, 9.8% (Wave 2) to 16.5% (Wave 1) of respondents had attempted to quit in the last year and 12.6% (Wave 2) to 22.8% (Wave 3) had plans to quit (table 2).

**Table 2:** Respondents’ unweighted baseline sociodemographic characteristics and tobacco behaviours

	Wave 1 (n=5991)	Wave 2 (n=5613)	Wave 3 (n=5636)
State			
Bihar	1696 (28.3%)	1646 (29.3%)	1497 (26.6%)
West Bengal	1077 (18%)	1069 (19%)	1092 (19.4%)
Madhya Pradesh	1459 (24.4%)	1377 (24.5%)	1472 (26.1%)
Maharashtra	1759 (29.4%)	1521 (27.1%)	1575 (27.9%)
Age group (years)			
15–17	158 (2.6%)	28 (0.5%)	120 (2.1%)
18–24	725 (12.1%)	587 (10.5%)	304 (5.4%)
25–39	1996 (33.3%)	1779 (31.7%)	1512 (26.8%)
40–54	1703 (28.4%)	1777 (31.7%)	1874 (33.3%)
>55	1409 (23.5%)	1357 (24.2%)	1551 (27.5%)
Not reported	0 (0.0%)	85 (1.5%)	275 (4.9%)
Sex			
Male	3439 (57.4%)	3260 (58.1%)	3289 (58.4%)
Female	2552 (42.6%)	2353 (41.9%)	2347 (41.6%)
Location			
Urban	4398 (73.4%)	4126 (73.5%)	4228 (75%)
Rural	1593 (26.6%)	1487 (26.5%)	1408 (25%)
Marital status			
Married	4321 (72.1%)	4110 (73.2%)	4054 (71.9%)
Widowed/separated/divorced	739 (12.3%)	681 (12.1%)	634 (11.2%)
Single	923 (15.4%)	813 (14.5%)	943 (16.7%)
Not reported	8 (0.1%)	9 (0.2%)	5 (0.1%)
Socioeconomic status			
Low	1321 (22%)	1240 (22.1%)	951 (16.9%)
Medium	2727 (45.5%)	2546 (45.4%)	2707 (48%)
High	1942 (32.4%)	1826 (32.5%)	1977 (35.1%)
Not reported	1 (0.0%)	1 (0.0%)	1 (0.0%)
Tobacco (SLT) Dependence Index			
0	1581 (26.4%)	1073 (19.1%)	1170 (20.8%)
1	1024 (17.1%)	822 (14.6%)	1081 (19.2%)
2	1801 (30.1%)	1756 (31.3%)	1601 (28.4%)
3	1191 (19.9%)	1490 (26.5%)	1023 (18.2%)
4	261 (4.4%)	227 (4%)	174 (3.1%)
5	53 (0.9%)	25 (0.4%)	17 (0.3%)
6	14 (0.2%)	10 (0.2%)	7 (0.1%)
Not reported	66 (1.1%)	210 (3.7%)	563 (10%)
Attempted to quit SLT in last year			
No	4968 (82.9%)	5062 (90.2%)	5050 (89.6%)
Yes	989 (16.5%)	550 (9.8%)	585 (10.4%)
Not reported	34 (0.6%)	1 (0%)	1 (0%)
Intentions to quit SLT			
No quit plans	5037 (84.1%)	4811 (85.7%)	4328 (76.8%)
Plan to quit	946 (15.8%)	706 (12.6%)	1286 (22.8%)
Not reported	8 (0.1%)	96 (1.7%)	22 (0.4%)

Wave 1=respondents reporting exclusive SLT use in Wave 1, Wave 2=respondents reporting exclusive SLT use in Wave 2, Wave 3=respondents reporting exclusive SLT use in Wave 3. Result was shown as Not reported when not every user had data for a given variable.

SLT, smokeless tobacco.

### SLT user’s responses to the HWL changes

Table 3 presents the results from the GEE-adjusted analysis for the seven HWL indicators.

**Table 3:** Results of generalised estimating equation models indicating the change in HWL effectiveness between waves

	Wave 1%(95% CI)	Wave 2%(95% CI)	Wave 3%(95% CI)	Wave 2–Wave 1		Wave 3–Wave 2		Wave 3–Wave 1		Test*	
Outcome measure				PP(95% CI)	P value	PP(95% CI)	P value	PP(95% CI)	P value	χ^2^	P value
All respondents											
Aware of warning labels on SLT packaging (n=8381, obs=16 166)	73.1(67.2 to 78.2)	73.4(67.4 to 78.6)	71.1(66.9 to 75.0)	0.3(−5.7 to 6.4)	0.909	−2.3(−9.0 to 4.4)	0.496	−1.9(−8.7 to 4.9)	0.570	0.51	0.775
Noticed warning labels ‘often/whenever use’ SLT (n=8368, obs=16 113)	37.2(30.7 to 44.1)	32.1(25.6 to 39.4)	35.8(31.2 to 40.5)	−5.1(−13.1 to 3.0)	0.213	3.6(−4.5 to 11.7)	0.373	−1.4(−9.9 to 7.0)	0.733	1.67	0.434
Among respondentswho noticed HWLs ‘often/whenever use’ SLT											
Read warning labels ‘often/regularly’ (n=4253, obs=5641)	50.8(43.5 to 58.0)	44.6(36.0 to 53.5)	32.8(27.7 to 38.5)	−6.2(−18.4 to 6.0)	0.313	−11.7(−22.0 to –1.5)	**0.026**†	−17.9(−27.4 to –8.4)	**<0.001**	15.92	**<0.001**
Warning labels make you think about risks ‘a lot’ (n=4229, obs=5605)	13.8(10.8 to 17.6)	17.9(11.9 to 26.0)	33.6(30.1 to 37.3)	4.1(−3.7 to 11.8)	0.296	15.7(7.4 to 24.0)	**<0.001**	19.8(14.8 to 24.8)	**<0.001**	52.09	**<0.001**
Warning labels make you ‘a lot’ more likely to quit (n=4216, obs=5573)	14.4(10.7 to 19.1)	18.9(12.8 to 27.0)	36.5(32.2 to 41.0)	4.5(−3.6 to 12.6)	0.274	17.6(8.8 to 26.4)	**<0.001**	22.1(15.1 to 29.0)	**<0.001**	35.45	**<0.001**
Avoided looking at warning labels (n=4246, obs=5632)	7.6(5.0 to 11.5)	10.1(7.0 to 14.5)	40.2(31.8 to 49.2)	2.5(−1.7 to 6.8)	0.238	30.1(21.1 to 39.0)	**<0.001**	32.6(22.5 to 42.6)	**<0.001**	58.96	**<0.001**
Gave up SLT ‘at least a couple of times’ because of warning labels (n=4237, obs=5618)	29(21.9 to 37.4)	36.6(27.5 to 46.8)	35.2(28.9 to 42.1)	7.6(−3.5 to 18.6)	0.175	−1.4(−14.1 to 11.3)	0.829	6.2(−4.7 to 17.1)	0.260	2.30	0.317

Estimates in columns ‘Wave 1’, ‘Wave 2’ and ‘Wave 3’ are predicted marginal estimates from the logistic model. Estimates in columns ‘Wave 2–Wave 1’, ‘Wave 3–Wave 2’ and ‘Wave 3–Wave 1’ are average marginal effects, or the percentage point difference between waves. Test.

* Test shows results of χ2 testing comparing results across all three waves. Tests do not account for multiple testing (although all significant results will remain significant if a Bonferroni correction is applied, except for the cell marked with a ‘†’). Covariates in logistic GEE models were survey wave, sex, age group, living in an urban area, marital status, socioeconomic status, past-year attempts to quit SLT (at least one vs none), intentions to quit SLT and Tobacco (SLT) Dependence Index.

GEE, generalised estimated equation; SLT, smokeless tobacco.

There were no significant changes in the percentage of respondents who were aware of HWLs across waves (Wave 1=73.1%, Wave 2=73.4%, Wave 3=71.1%, p=0.78) and who noticed HWLs ‘often’ between waves (Wave 1=37.2%, Wave 2=32.1%, Wave 3=35.8%, p=0.43). Among respondents who noticed HWLs often, there was a decrease in reading the HWLs ‘often’ or ‘regularly’ (Wave 1=50.8%, Wave 2=44.6%, Wave 3=32.8%, p<0.001).

Cognitive reactions to HWLs improved across waves. Of those who noticed HWLs, there was a significant increase in the percentage who thought about SLT risks ‘a lot’ between Waves 1 (13.8%), 2 (17.9%) and 3 (33.6%) (p<0.001). Additionally, there was a significant increase in the percentage of respondents who were ‘a lot’ more likely to quit SLT use because of HWLs between Waves 1 (14.4%), 2 (18.9%) and 3 (36.6%) (p<0.001).

Behavioural outcomes showed mixed results. Of those who noticed HWLs, there was a significant increase in the percentage who avoided looking at HWLs between Waves 1 (7.6%), 2 (10.1%) and 3 (40.2%) (p<0.001). There was no significant difference in the percentage of respondents who did not use SLT due to HWLs between Waves 1 (29.0%), 2 (36.6%) and 3 (35.2%) (p=0.32).

### Health warning labels effectiveness after the 2011 SLT policy change

Following the change from a symbolic to a pictorial HWL, there were no significant differences in HWL effectiveness outcomes between Waves 1 and 2 (all p≥0.05)

### Health warning label effectiveness after the 2016 SLT policy change

There was no significant change in the percentage of individuals who were aware of HWLs (Wave 2=73.4%, Wave 3=71.1%, p=0.91), nor in the percentage who noticed them ‘often’ or ‘regularly’ (Wave 2=37.2%, Wave 3=35.8%, p=0.21). Of those who noticed HWLs, following corrections for multiple comparisons, there was no significant change in the percentage who read HWLs ‘often’ or ‘regularly’ (Wave 2=44.6%, Wave 3=32.8%, Bonferroni adjusted p=0.08). There were large increases in the percentage that thought about tobacco risks ‘a lot’ due to HWLs (Wave 2=17.9%, Wave 3=33.6%, p<0.001), who were ‘a lot’ more likely to quit SLT use due to HWLs (Wave 2=18.9%, Wave 3=36.6%, p<0.001), and who avoided looking at HWLs (Wave 2=10.1%, Wave 3=40.2%, p<0.001). There was no significant change in the percentage who gave up SLT ‘at least a couple of times’ because of HWLs (Wave 2=36.6%, Wave 3=35.2%, p=0.83).

### Predictors of quitting SLT

Table 4 shows results from logistic GEE regression models examining factors predictive of quitting SLT by the follow-up survey wave. Among all respondents, HWL effectiveness, as measured by LII, was not associated with quitting, and this did not differ by baseline wave. Respondents who were single, female, had higher SES and were from Maharashtra (compared with Bihar Respondents) were more likely to quit SLT use. Making a previous attempt to quit SLT was not predictive of quitting at follow-up, whereas having intentions to quit and lower SLT dependence were strongly associated with quitting.

**Table 4:** Logistic regression analysis examining predictors of quitting SLT

	aOR (95% CI)
Labels Impact Index (LII)	
1 unit increase	1.02 (0.99 to 1.05)
Wave LII interaction	
Wave 2 (vs Wave 1), 1 unit increase in LII (interaction OR)	0.98 (0.93 to 1.02)
Wave 2, 1 unit increase in LII	1.02 (0.99 to 1.05)
Wave 1, 1 unit increase in LII	0.99 (0.96 to 1.03)
State	
Maharashtra (vs Bihar)	2.15 (1.31 to 3.52)
Madhya Pradesh (vs Bihar)	1.62 (0.88 to 2.98)
West Bengal (vs Bihar)	0.98 (0.57 to 1.67)
Urban/rural	
Rural (vs urban)	1.38 (0.86 to 2.19)
Sex	
Male (vs female)	0.55 (0.42 to 0.71)
Age group (years)	
55+ (vs 15–17)	0.91 (0.51 to 1.61)
40–54 (vs 15–17)	0.61 (0.35 to 1.06)
25–39 (vs 15–17)	0.88 (0.49 to 1.56)
18–24 (vs 15–17)	1.06 (0.62 to 1.84)
Marital status	
Single (vs married)	1.44 (1.07 to 1.95)
Widowed/divorced/separated (vs married)	0.98 (0.68 to 1.42)
Socioeconomic status	
Low (vs high)	0.5 (0.35 to 0.72)
Moderate (vs high)	0.73 (0.59 to 0.92)
Attempts to quit in past year	
At least one attempt (vs none)	1.01 (0.74 to 1.37)
Intentions to quit	
Plans to quit (vs no plans)	1.37 (1.03 to 1.83)
Wave	
Wave 2 (vs Wave 1)	1.19 (0.82 to 1.74)
Level of Tobacco (SLT) Dependence Index	
Dependence (1 unit increase)	0.79 (0.71 to 0.88)

Estimates show the adjusted OR for each variable. ORs show the effect of each variable at the initial wave (Wave one or Wave 2) in predicting whether a respondent quit tobacco by the following wave (Waves 2 and 3 respectively). Covariates included state, living in an urban/ rural area, sex, age, marital status, socioeconomic status, attempts to quit in past year, intentions to quit, wave effects dependence, LII and LII*wave interactions.

## Discussion

This study aimed to assess the effectiveness of both the old (symbolic, smaller pictorial) and new (larger pictorial) HWLs in India, as well as the impact of old HWL effectiveness on quitting SLT use in a group of respondents who use SLT. The 2011 transition from a symbolic image of a scorpion to a pictorial image, both covering 40% of tobacco packaging did not significantly change HWL effectiveness in individuals who use SLT, supporting previous research.^20^ Effectiveness of older (smaller symbolic and smaller pictorial) HWLs was not associated with quitting SLT. The enlargement of HWLs in 2016 was associated with increased effectiveness for some cognitive and behavioural outcomes but was not with changes in HWL awareness or salience. An examination of the impact of these more effective, enlarged HWLs on SLT cessation is urgently needed.

Results regarding the impact of increased HWL size were mixed. Such findings on resultant HWL awareness, salience, cognitive effects and behavioural effects draw parallels and contrasts in the literature. Results corroborated previous findings of improved cognitive effects following increased HWL size.^21 26^ Despite these changes over time, only 33.6% of respondents reported that the larger HWLs made them think about risks ‘a lot’ and 36.5% that HWLs made them ‘a lot’ more likely to quit. With regard to behavioural outcomes, we found mixed results. Consistent with previous research, respondents were more likely to avoid HWLs.^21 26^ However, there was no significant change in HWLs stopping an individual from using tobacco, in contrast with the effects of increased HWL size on cigarette packaging in Uruguay.^21^ Our study used a different cut-off, where HWLs had to stop respondents using tobacco ‘a lot’, rather than ‘at least a couple of times’. Additionally, the number of respondents reporting such an effect in Uruguay smokers was very low (1.9% before vs 6.1% following change). These results align with findings following increased cigarette HWL size in Australia, where there was no change in this measure.^26^ It may be that at the time of tobacco use, when craving is maximal, HWLs and their size may not strongly prevent tobacco consumption.

HWL awareness did not change across survey waves, even following the increased size. These results confirm those of other studies in India whereby HWLs have low levels of HWL awareness.^16 33^ Lack of change in awareness may be attributed to factors that prevent individuals from encountering HWLs, for example, limited manufacturer compliance with the regulations,^34^,^36^ illicit SLT and the purchase of loose rather than packaged SLT products.^24^ A study that collected SLT packages following increased HWL size requirements reported that while 97.5% of Indian SLT products had an HWL, compliance with all regulations was poor. For example, 12% only covered between 25% and 49% of the packet’s surface.^37^ Additionally, SLT packaging designs can effectively counteract warning content through creative techniques that undermine the salience and impact of the warnings.

Similarly, there was no increase in noticing or reading HWLs following the size increase. Moreover, reading of HWLs significantly decreased between Waves 1 and 3. Measures of reading may have been affected by the inclusion of Hindi rather than the previous sole use of English on HWLs. Internationally, while some studies have found that increased HWL size increases salience,^13 21^ the increase in size of Australian cigarette HWLs also had no effect on the frequency that a respondent read an HWL.^38^ This was partly attributed to some respondents learning to systematically avoid focusing their attention on HWLs. Additionally, it was suggested that as the graphic picture tells the story, the text may not be read as often. Alternatively, decreased reading of HWLs may indicate that new HWLs are more unattractive, which may positively impact subsequent quit attempts. Additionally, HWL wear-out results in diminished HWL effectiveness over time, requiring HWLs to be changed regularly.^30 39^ Given both the small number of unique HWLs and that, in this study, baseline respondents answered the questionnaires when HWLs were novel, this effect becomes particularly relevant. Contrasting results, where respondents who use smoked tobacco in India noticed HWLs more in 2016–2017 compared with 2009–2010,^24^ strengthen the argument of wear-out having occurred, given that the follow-up interview of these individuals occurred soon after increased HWL size requirement.

While the Indian government’s ambitious HWL legislation should be lauded, these results appear to suggest that further efforts are needed to strengthen warnings on SLT packaging, including refreshing warnings more regularly to prevent wear-out. Moreover, efforts to combat illicit products and non-compliant SLT packaging are urgently needed.

### Strengths and limitations

This is the first study to assess the effectiveness of new SLT HWLs across time in India. Despite respondents being lost to attrition due to a long follow-up time, a large number of respondents were included in the analysis. As respondents were only sampled from four Indian states, caution is needed when generalising results across India as a whole. The sample included only people who used only SLT and not those who used both SLT and smoked tobacco; however, people who used both products were excluded to prevent contamination of results from those exposed to HWLs on smoked tobacco and SLT products. The LII has previously only been used in people using smoked tobacco, given the higher prevalence of smoking globally. Additionally, some individuals may have lower LII scores not due to the HWL ineffectiveness per se, but rather due to a lack of HWL implementation, which may have limited HWL awareness. Similarly, HWL non-compliance and purchase of loose SLT limits measures of HWL effectiveness, as not all SLT an individual encounters may be fully in accordance with regulations. This analysis did not assess the effect of HWLs in dissuading people who do not use tobacco from starting to use tobacco, which may be a mechanism by which they are effective in reducing tobacco prevalence. The analysis used a more accurately measured index of SLT dependence compared with previous studies. Finally, the results may be subject to recall bias, and possibly social desirability as the surveys were conducted in person.

## Conclusion

Between 2009 and 2016, the Indian government implemented ambitious legislation to strengthen HWLs. Over this time. there have been no positive changes in HWL awareness and salience, but among those who interacted with HWLs, there was some improvement for some cognitive and behavioural reactions after the HWL was enlarged. Such evidence supports the actions of other countries in increasing HWL size on tobacco packaging and gives evidence of the relative effect of symbolic and pictorial HWLs. This study uses a Tobacco Dependence Index, shown to be strongly predictive of quitting SLT, appropriate to the future study of SLT control in India. This positive finding can be strengthened by a range of actions by researchers and policymakers, especially by preventing barriers that limit HWL awareness, salience and wear-out.

## Data Availability

Data are available upon reasonable request.
